# Identification of Bexarotene as a PPAR**γ** Antagonist with HDX

**DOI:** 10.1155/2015/254560

**Published:** 2015-09-15

**Authors:** David P. Marciano, Dana S. Kuruvilla, Bruce D. Pascal, Patrick R. Griffin

**Affiliations:** Department of Molecular Therapeutics, The Scripps Research Institute, Scripps Florida, Jupiter, FL 33458, USA

## Abstract

The retinoid x receptors (RXRs) are the pharmacological target of Bexarotene, an antineoplastic agent indicated for the treatment of cutaneous T cell lymphoma (CTCL). The RXRs form heterodimers with several nuclear receptors (NRs), including peroxisome proliferator-activated receptor gamma (PPAR*γ*), to regulate target gene expression through cooperative recruitment of transcriptional machinery. Here we have applied hydrogen/deuterium exchange (HDX) mass spectrometry to characterize the effects of Bexarotene on the conformational plasticity of the intact RXR*α*:PPAR*γ* heterodimer. Interestingly, addition of Bexarotene to PPAR*γ* in the absence of RXR*α* induced protection from solvent exchange, suggesting direct receptor binding. This observation was confirmed using a competitive binding assay. Furthermore, Bexarotene functioned as a PPAR*γ* antagonist able to alter rosiglitazone induced transactivation in a cell based promoter:reporter transactivation assay. Together these results highlight the complex polypharmacology of lipophilic NR targeted small molecules and the utility of HDX for identifying and characterizing these interactions.

## 1. Introduction

The retinoid x receptors (RXR*α*, *β*, and *γ*) form heterodimers with a subclass of nuclear receptors (NRs) that include PPARs, LXRs, FXRs, PXRs, RARs, CAR, TR, and VDR to cooperatively modulate gene expression [1, 2]. These heterodimers can be classified as permissive, whereby agonists for either heterodimer partner can activate gene expression, or nonpermissive for which RXR agonists alone have no effect on transcriptional activity but can synergistically induce hyperactivation with partner agonists [3, 4]. The structural determinants delineating permissive and nonpermissive RXR heterodimers have been the focus of significant study, as heterodimer-selective retinoids may hold therapeutic potential for the treatment of metabolic disease [5].

Peroxisome proliferator-activated receptor gamma (PPAR*γ*) is a permissive RXR heterodimer partner [6] and the pharmacological target of the thiazolidinedione (TZD) class of insulin sensitizers that include rosiglitazone [7]. RXR targeted retinoids have also been demonstrated to act as insulin sensitizers in rodent models [8], through what appear to be both conserved and unique mechanisms [9, 10]. While the TZDs have been shown to increase body weight and fat mass in both rodents and humans (and perhaps food intake in rodents), retinoids, partly mediated by the CNS, reduce food consumption and decrease body weight and fat mass in rodents [11, 12]. It is interesting to note that a similar phenotype has been reported with pharmacological PPAR*γ* repression in the CNS [13].

Bexarotene (Targretin, formerly LGD1069) is a third generation retinoid antineoplastic agent that potently activates RXRs [14] and is approved for the treatment of cutaneous T cell lymphoma (CTCL) [15]. Bexarotene potently activates adipocyte differentiation in multipotent mesenchymal stromal cells but with 20% maximal efficacy relative to PPAR*γ* agonist rosiglitazone [16]. To characterize the effects of Bexarotene binding to RXR*α* on the conformational plasticity of its permissive coreceptor PPAR*γ*, we applied hydrogen-deuterium exchange (HDX) coupled with mass spectrometry to analyze the intact heterodimer. These studies revealed that Bexarotene directly binds to PPAR*γ* at functionally relevant concentrations. Additional studies demonstrate that Bexarotene functions as a PPAR*γ* antagonist. The results presented here highlight the complex polypharmacology of lipophilic small molecules targeting nuclear receptors and the utility of HDX in characterizing these interactions.

## 2. Materials and Methods

### 2.1. HDX-MS

Solution-phase amide HDX experiments were carried out using a fully automated system as described previously [17]. The PPAR*γ* and RXR*α* LBDs were expressed and purified as previously reported [18]. 10 *μ*M of PPAR*γ* and RXR*α* LBD protein (20 mM KPO_4_, pH 7.4, 50 mM KCl) was preincubated with 1 : 2 molar excess of compound or DMSO control. 5 *μ*L of protein solution was mixed with 20 *μ*L of D_2_O-containing HDX buffer (20 mM KPO_4_, pH 7.4, 50 mM KCl) and incubated at 4°C for 10 s, 30 s, 60 s, 900 s, and 3,600 s. Following on-exchange, unwanted forward or back exchange was minimized and the protein was denatured by dilution with 25 *μ*L of quench solution (0.1% v/v TFA in 3 M urea). Samples were then passed through an immobilized pepsin column at 200 *μ*L min^−1^ (0.1% v/v TFA, 15°C) and the resulting peptides were trapped on a C_8_ trap column (Hypersil Gold, Thermo Scientific, CA). The bound peptides were then gradient-eluted (5–50% CH_3_CN w/v and 0.3% w/v formic acid) across a 2 mm × 50 mm C_18_ HPLC column (Hypersil Gold, Thermo Scientific, CA) for 5 min at 4°C. The eluted peptides were then subjected to electrospray ionization directly coupled to a high resolution Orbitrap QExactive mass spectrometer (Thermo Scientific, CA). Each HDX experiment was carried out in triplicate and the intensity weighted average* m/z* value (centroid) of each peptide isotopic envelope was calculated with in-house HDX Workbench software [19].

### 2.2. PPAR*γ* Binding Assay

PPAR*γ* competitive binding assay (Invitrogen) was performed according to the manufacturer's protocol. A mixture of 5 nM glutathione *S*-transferase fused with human PPAR*γ* ligand binding domain (GST–PPAR*γ*–LBD), 5 nM Tb-GST-antibody, 5 nM Fluormone Pan-PPAR Green, and serial dilutions of compound beginning at 10 *μ*M downwards was added to wells of black 384-well low-volume plates (Greiner) to a total volume of 18 *μ*L. All dilutions were made in TR-FRET PPAR assay buffer. DMSO at 2% final concentration was used as a no-ligand control. Experiments were performed in triplicate and incubated for 2 h in the dark before analysis in Perkin Elmer ViewLux ultra HTS microplate reader. The FRET signal was measured by excitation at 340 nm and emission at 520 nm for fluorescein and 490 nm for terbium. The fold change over DMSO was calculated by 520 nm/490 nm ratio. Graphs were plotted in GraphPad Prism (La Jolla, CA) as fold change of compound FRET signal over DMSO-only control and EC_50_ calculated.

### 2.3. Cell Based Transactivation Assay

HEK293T cells (ATCC; cat# CRL-3216) were cotransfected in batch by adding 4.5 *μ*g human PPAR*γ*2-Gal4, with 4.5 *μ*g UAS-luciferase reporter and 27 *μ*L X-treme Gene 9 transfection reagent in serum-free Opti-mem reduced serum media (Gibco). After 18-hour incubation at 37°C in a 5% CO_2_ incubator, transfected cells were plated in quadruplicate in white 384-well plates (Perkin Elmer) at a density of 10,000 cells per well. After replating, cells were treated with either DMSO only or the indicated compounds in increasing doses from 2 pM to 10 *μ*M. After 18-hour incubation, treated cells were developed with Brite Lite Plus (Perkin Elmer) and read in 384-well Luminescence Perkin Elmer EnVision Multilabel Plate Reader. Graphs were plotted as fold change of treated cells over DMSO-treated control cells.

## 3. Results

**Figure 1:**
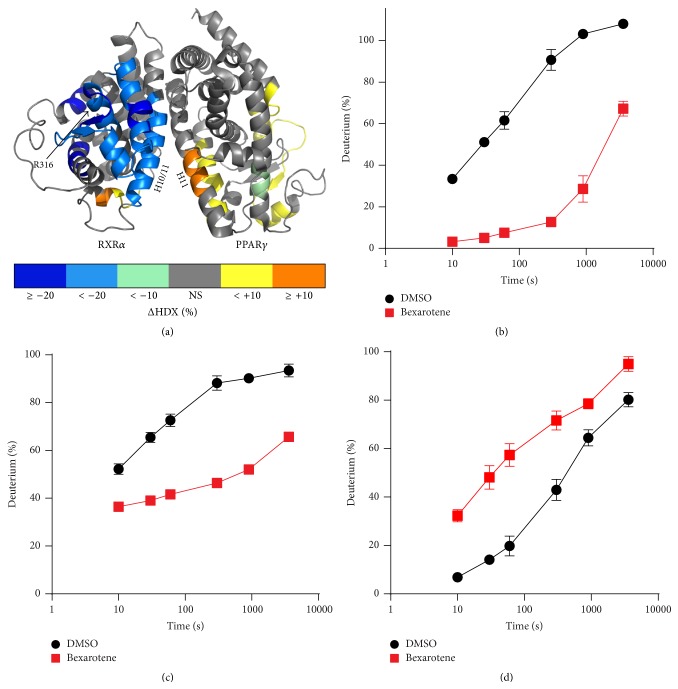
Differential HDX of PPAR*γ*:RXR*α* heterodimer with Bexarotene: (a) residues colored corresponding to the average percent change in deuteration between apo and Bexarotene bound complex over 6 time points (10, 30, 60, 300, 900, and 3600 seconds) run in triplicate (*n* = 3) overlaid on PDB:1 K74. HDX buildup curves of (b) RXR*α* helix 10/11 peptide (RSIGLKC) at the dimer interface, (c) RXR*α* peptide (SHRSIAVKDGIL) containing arginine 316 known to form a hydrogen bond with Bexarotene in crystal structure PDB 4K61, and (d) PPAR*γ* LBD helix 11 peptide (RQIVTEHVQL) at dimer interface.

To characterize the allosteric effects of ligand binding to RXR*α* on the conformational plasticity of PPAR*γ*, differential HDX was applied to study the intact complex (Figure 1(a)). Addition of Bexarotene resulted in significant protection throughout the RXR*α* ligand binding domain (Figures 1(b) and 1(c)), consistent with high affinity receptor binding [20]. In contrast, several regions of the PPAR*γ* LBD demonstrated increased exchange including a region at the dimer interface (Figure 1(d)). These data suggest that Bexarotene allosterically alters the conformational dynamics of the PPAR*γ* coreceptor upon binding to RXR*α*.

**Figure 2:**
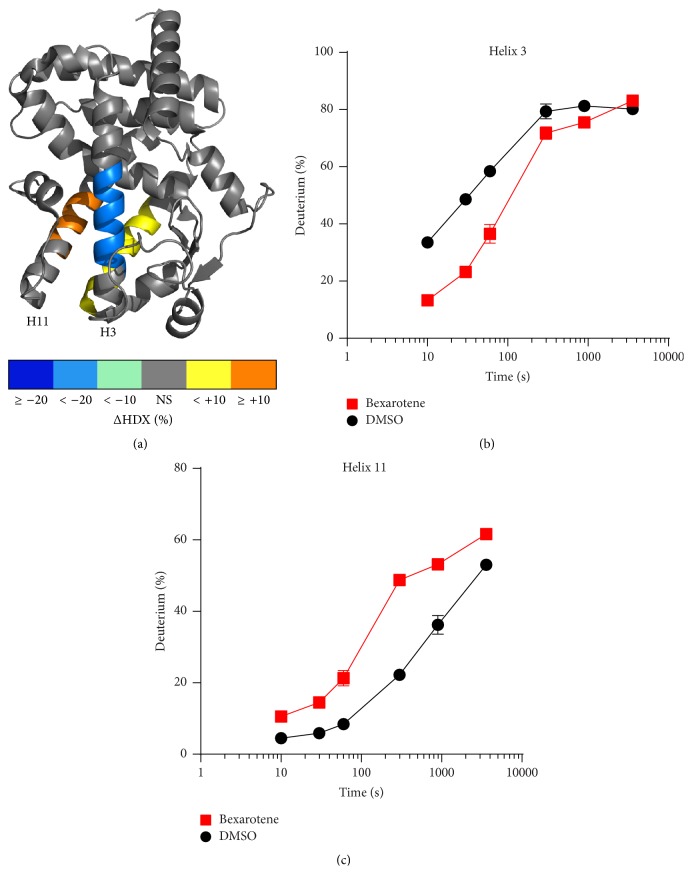
Differential HDX of PPAR*γ* with Bexarotene: (a) residues colored corresponding to the average percent change in deuteration between apo and Bexarotene bound PPAR*γ* over 6 time points (10, 30, 60, 300, 900, and 3600 seconds) run in triplicate (*n* = 3) overlaid on PDB:1K74. HDX buildup curves of (b) PPAR*γ* LBD helix 3 peptide IRIFQGCQ (blue) and (c) PPAR*γ* LBD helix 11 RXIVTEHVQL (orange).

**Figure 3:**
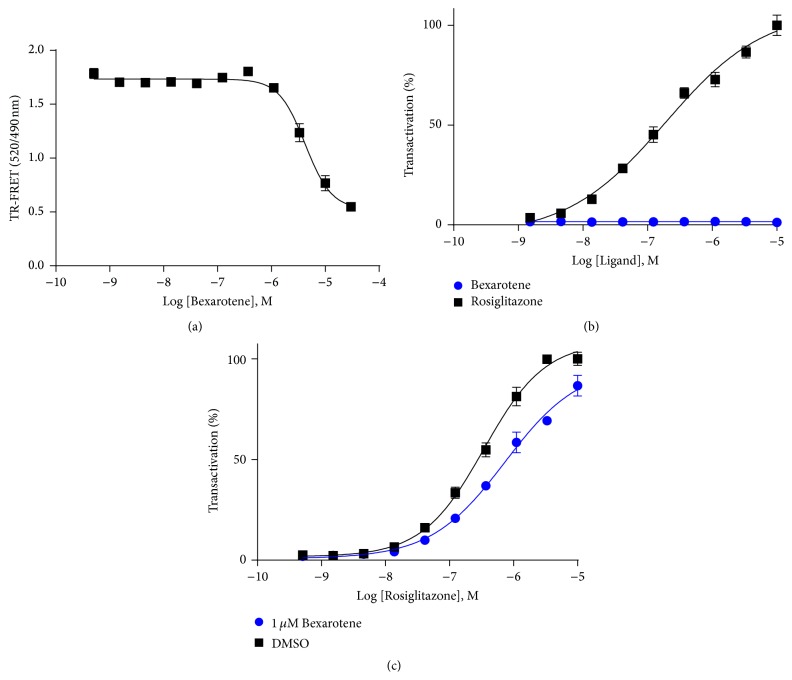
Biochemical characterization of Bexarotene on PPAR*γ*: (a) TR-FRET competitive displacement assay (*n* = 3). (b) Dose dependent transcriptional activity of a PPAR*γ*:PPRE-luciferase promoter-reporter assay in HEK293T cells (*n* = 4). (c) Dose dependent transcriptional activity of rosiglitazone ±1 *μ*M Bexarotene in a PPAR*γ*:PPRE-luciferase promoter-reporter assay in HEK293T cells (*n* = 4).

To confirm that the alterations in HDX kinetics observed on PPAR*γ* were indeed allosteric, HDX analysis of PPAR*γ* alone in the presence and absence of Bexarotene was performed. Surprisingly, addition of Bexarotene to PPAR*γ* alone altered deuterium exchange kinetics similar to that observed in analysis of ligands known to directly bind PPAR*γ*, including similar protection to exchange on helix 3 (Figures 2(a) and 2(b)) [21]. Notably, Bexarotene had no effect on helix 12 deuterium incorporation (Figure 2(c)), mirroring the HDX profile of SR1664, a known PPAR*γ* antagonist [18]. To confirm direct binding of Bexarotene to PPAR*γ*, a TR-FRET competitive displacement assay was performed demonstrating an IC_50_ ~ 3 *μ*M (Figure 3(a)). A cotransfection promoter:reporter gene assay was performed, and the results revealed that Bexarotene alone cannot transactivate the reporter gene (Figure 3(b)). However, in a competitive assay, Bexarotene right shifted the EC_50_ of rosiglitazone mediated reporter gene transactivation (Figure 3(c)), confirming that it binds directly to PPAR*γ* and functions as an antagonist.

## 4. Discussion

The strategy of repurposing pharmaceuticals has emerged in response to the challenges and expense of obtaining regulatory approval for new drugs [22, 23]. Drug repurposing is particularly common in personalized cancer treatments, where tumors are screened for aberrant pathways to rationally intervene with appropriate therapies. An important compliment to expand the reach of already approved drugs is to characterize their complex polypharmacology and drug interactomes. Nuclear receptor pharmacology efforts to date have focused primarily on subtype selectivity for preferential isoform targeting [24, 25]. While this remains an important consideration, it has become apparent that the polypharmacology of NR targeted lipophilic small molecules spans the entire superfamily and beyond [26, 27]. This will be an important consideration with the emerging focus on delineating closely related ligands to improve therapeutic index using pathway analysis, particularly with the expanded repertoire of complexity now appreciated for nuclear receptor signaling [28]. While screening kinase panels has become requisite in the development of novel inhibitors [29], this has yet to become routine for nuclear receptor pharmacology despite the homology of ligand binding domains and redundancy in endogenous ligands [30, 31]. HDX is well-positioned to interrogate* in vitro* pharmacomic interactions with the advent of automated platforms and data processing software compatible with requisite screening throughputs [32].

Bexarotene is approved for the treatment of CTCL and, like most chemotherapies, has been investigated for efficacy in other cancer types [33]. Bexarotene has also been reported to reduce amyloid plaque and improve mental function in the APP/PS1 Alzheimer's mouse model [34], with clinical trials ongoing to determine whether this will translate to man. Here we have demonstrated off-target binding of Bexarotene to PPAR*γ*, also a target that has emerged for the treatment of Alzheimer's disease [35]. A systematic analysis of the interactome for these promising repurposing candidates will be important in identifying the true mechanism of action along with minimizing off-target adverse effects. For example, the off-target affinity of Bexarotene for PPAR*γ* may contribute to the reported insulin-sensitizing efficacy of retinoids through modulation of receptor posttranslational modifications [28].

## 5. Conclusion

Here we have applied HDX to identify the off-target binding of Bexarotene to PPAR*γ* and confirmed this with a competitive binding assay. Bexarotene acts as a PPAR*γ* antagonist in a cell based promoter:reporter transactivation assay, competing with rosiglitazone, and has a HDX profile consistent with other known PPAR*γ* antagonists. The ability of Bexarotene to modulate PPAR*γ* may contribute to the beneficial effects observed in animal models of insulin resistance and Alzheimer's disease. Together these results highlight the complex polypharmacology of NR ligands, the utility of HDX in characterizing these interactions, and the importance of characterizing ligands across the NR superfamily.
